# Continuity and breaches in GP care and their associations with mortality for patients with chronic disease: an observational study using Norwegian registry data

**DOI:** 10.3399/BJGP.2023.0211

**Published:** 2024-04-16

**Authors:** Sahar Pahlavanyali, Øystein Hetlevik, Valborg Baste, Jesper Blinkenberg, Steinar Hunskaar

**Affiliations:** Department of Global Public Health and Primary Care, University of Bergen, Bergen.; Department of Global Public Health and Primary Care, University of Bergen, Bergen.; The National Centre for Emergency Primary Health Care, NORCE Norwegian Research Centre, Bergen.; The National Centre for Emergency Primary Health Care, NORCE Norwegian Research Centre, Bergen.; Department of Global Public Health and Primary Care, University of Bergen; head, The National Centre for Emergency Primary Health Care, NORCE Norwegian Research Centre, Bergen.

**Keywords:** chronic disease, continuity of care, general practice, mortality, observational study, primary health care

## Abstract

**Background:**

Despite many benefits of continuity of care with a named regular GP (RGP), continuity is deteriorating in many countries.

**Aim:**

To investigate the association between RGP continuity and mortality, in a personal list system, in addition to examining how breaches in continuity affect this association for patients with chronic diseases.

**Design and setting:**

A registry-based observational study using Norwegian primary care consultation data for patients with asthma, chronic obstructive pulmonary disease (COPD), diabetes mellitus, or heart failure.

**Method:**

The Usual Provider of Care (UPC, value 0–1) Index was used to measure both disease-related (UPC^disease^) and overall (UPC^all^) continuity with the RGP at the time of consultation. In most analyses, patients who changed RGP during the study period were excluded. In the combined group of all four chronic conditions, the proportion of consultations with other GPs and out-of-hours services was calculated. Cox regression models calculated the associations between continuity during 2013–2016 and mortality in 2017–2018.

**Results:**

Patients with COPD with UPC^disease^ <0.25 had 47% increased risk of dying within 2 years (hazard ratio 1.47, 95% confidence interval = 1.22 to 1.64) compared with those with UPC^disease^ ≥0.75. Mortality also increased with decreasing UPC^disease^ for patients with heart failure and decreasing UPC^all^ for those with diabetes. In the combined group of chronic conditions, mortality increased with decreasing UPC^all^. This latter association was also found for patients who had changed RGP.

**Conclusion:**

Higher disease-related and overall RGP UPC are both associated with lower mortality. However, changing RGP did not significantly affect mortality, indicating a compensatory benefit of informational and management continuity in a patient list system.

## Introduction

There is a general perception that continuity of care (CoC) in general practice is linked to increased use of preventive care,^1^ better medication adherence,^2^^,^^3^ reduced polypharmacy,^4^ lower utilisation of health care,^5^ fewer acute hospital admissions,^6^^,^^7^ and lower mortality.^6^^,^^8^^,^^9^ Despite this, continuity is declining in many countries,^10^^–^12 and structural changes in healthcare systems, prioritising quick access to any GP, and increasing GP workload are discussed as common reasons.^13^^,^^14^ A system with GP personal lists might be one of the measures considered to prevent declining personal continuity, defined as a therapeutic relationship between a patient and a GP over time.^15^ A named GP is also a home for patients’ medical history, and thus tracks and helps to coordinate patient care. In addition to facilitating personal continuity, a personal list assigned to a named GP ensures continuity of information (access to patient’s health records) and management (consistent and coordinated care delivered by several providers),^9^^,^^15^^,^^16^ even when a different GP acquires the role of being the patient’s regular GP (RGP) by taking over the personal list.

In Norway, approximately 15% of the population change their RGP yearly, either at the patients’ discretion or because GPs leave or retire.^17^ It is not known to what extent a well-organised patient list system with a clear role for the RGP might compensate for possible negative effects of such planned or unplanned breaches in personal continuity. But even in a well-functioning patient list system the CoC will be somewhat affected when necessary consultations are done both by other GPs because of unavailability of the patient’s RGP, and because of out-of-hours (OOH) appointments. The effects of such breaches of CoC are also sparsely investigated.

Research claims that patients with chronic diseases benefit from all types of CoC,^18^^–^22 although evidence is limited on whether patients benefit from disease-related or overall CoC. In a previous study on patients with asthma, chronic obstructive pulmonary disease (COPD), diabetes mellitus, and heart failure, this author group demonstrated lower mortality associated with higher chronic care continuity in the overall healthcare system.^23^

**Table:** How this fits in

There is a growing body of evidence on the advantages of continuity, and a GP personal list system is believed to be one of the positive measures to improve continuity, although not much researched. In this study, in a Norwegian setting with GP personal lists, the associations between GP continuity and mortality for patients with different chronic diseases was investigated. The results showed that lower GP continuity was associated with increased risk of death, but the association was not significantly different for patients with the same named regular GP (RGP) compared with those with different RGPs. This study suggests that high informational and management continuity provided by a GP personal list might lower and compensate for adverse effects when changing GP.

This study aimed to extend current knowledge about the association between CoC with a named RGP and mortality by comparing CoC related to care for four specific chronic conditions, with CoC based on all care given by the same RGP for these patients within the study period. Further, how breaches in personal continuity after changing named RGP affected the association between CoC and mortality was investigated. Finally, whether the association between CoC and mortality was affected by different causes of discontinuity was examined — for example, consultations with other GPs or OOH services.

## Method

### Norwegian primary health care

Norwegian general practices and OOH services are managed by municipalities as a part of primary health care. The RGP scheme allocates a personal RGP to nearly the entire Norwegian population (>99%).^17^ Patients are allowed to change RGP twice a year. The practices mostly consist of 3–6 RGPs working together with common electronic patient records.^6^ OOH services are staffed with primary care doctors who are used for immediate medical assistance or care out of office hours.

### Design and data sources

In this longitudinal observational study, pseudo-anonymised data were provided by the following registries.

- The Control and Payment of Reimbursement to Health Service Providers database (KUHR) provided consultations and home visits data from RGPs and OOH services. For each contact ≥1 diagnoses from The International Classification of Primary Care, second edition (ICPC-2) are registered.
- The Norwegian Patient Registry (NPR) provided consultation data from private specialists with a public contract, hospital outpatient clinics, data on hospital admissions, and some data from OOH services. For each contact ≥1 International Classification of Diseases (tenth edition) diagnoses are recorded.
- The Norwegian RGP registry supplied information about each patient’s RGP.
- Statistics Norway (SSB) provided the study with date of birth, sex, date of death, centrality classes (urban/rural),^24^ and educational levels.^25^

SSB linked data from these registries by using a pseudo-id for each patient’s national identification number.

### Study population

All patients with ≥1 consultation with a diagnosis of asthma, COPD, diabetes mellitus, or heart failure within the registries during 2012 were included. For all these patients, data were collected on all consultations and home visits with the patient’s RGP, other GPs, and OOH services during 2013–2016.

The patients were categorised into five study populations:

- four disease-specific populations that were not mutually exclusive for those who had ≥2 disease-related consultations for the respective diagnosis and had the same RGP during 2013–2016; and
- one combined population across these disease-specific groups of patients with ≥2 all-cause consultations in 2013–2016, named ‘the group of chronic conditions’, also including patients who had changed their RGP during the study period.

The following were excluded from all the groups: those who died by 31 December 2016; patients with no primary care consultations in 2013–2016; and those with no consultations with an RGP practice in 2016, the latter to exclude patients with long-term stays in nursing homes (not reported in the KUHR database).

### CoC

CoC was measured by the Usual Provider of Care (UPC) Index,^26^ well suited for claims-based data investigating density of contacts with one provider^27^ and a proxy for personal continuity, but not a direct measure. The UPC is calculated as *n/N*, where *n* is the total number of consultations with the usual provider, in this study referring to the named RGP at the time of consultations, and *N* represents total number of consultations in primary care including other GPs and OOH services. UPC was calculated in this study in two different ways for the populations with asthma, COPD, diabetes mellitus, and heart failure, first based on only disease-related consultations (UPC^disease^) and second based on all consultations (UPC^all^). For the group of chronic conditions, UPC was calculated based on all consultations. For patients who had changed their RGP during 2013–2016, the UPC reflects the proportion of consultations with the RGP responsible for the personal list at the time of consultation. For the group of chronic conditions, the proportions of consultations with other GPs (this includes other GPs in the same practice, locum doctors, and interns) was also calculated as the Other GPs Index and proportions of all OOH services consultations called OOH Index, representing different breaches of CoC. These analyses were undertaken only for those with the same RGP during the study period.

UPC values are on a scale of 0 to 1 (1 = all consultations by the RGP). In this study, for these analyses the values were divided into four categories: <0.25, 0.25–<0.50, 0.50–<0.75, and 0.75–1. For Other GP Index (<0.1, 0.1–<0.2, 0.2–<0.3, 0.3–<0.4, and ≥0.4) and OOH Index (<0.05, 0.05–<0.1, 0.1–<0.15, 0.15–<0.2, and ≥0.2) five categories were used.

### Mortality

SSB data were used to identify the date of death for those who died in 2017 and 2018. The authors did not have access to cause of death.

### Study covariates

The following covariates were used: patient’s age (continuous), sex, Centrality Index, educational level, number of comorbidities, and number of consultations with the RGP, other GPs, and OOH services, both for consultations related to the four diagnoses and all consultations in the group of chronic conditions. For the latter, the authors also defined a dichotomic variable indicating same or change of RGP, ‘same RGP’.

The municipalities are classified into six levels (Centrality Index) by SSB, level one including the most central (urban) areas and level six the least central (rural) areas.^24^ Highest level of education is categorised as low (elementary school or less), medium (upper secondary school), and high (university and higher education).^25^ For comorbidities the ICPC Morbidity Index^28^ was used divided into four groups (0, 1, 2, and ≥3 comorbidities).

### Statistical analyses

Descriptive statistics included frequencies, percentages, means, and standard deviations (SDs). To investigate the associations between CoC and mortality, Cox regression was performed for each of the five populations separately. Time to death was calculated in days from 1 January 2017, and the follow-up time was 24 months or until death. UPC (four categories) was the independent variable with the highest UPC category as reference. Hazard ratios (HRs) and 95% confidence intervals (CIs) were calculated and both crude and adjusted analyses were performed. In the diagnosis populations different models were run using UPC^disease^ and UPC^all^ as predictors.

In the analyses of the group of chronic conditions an interaction term between same RGP and UPC was tested in the Cox regression model, and the results were presented stratified by same RGP. Educational level was not included in the adjusted models as this variable had 19.3% (*n* = 4634/23 982) missing values for asthma and there were marginal changes in the adjusted results. Other covariates had very few missing data (<0.01%).

Additionally, separate adjusted Cox regression models were used to investigate the association between the Other GPs Index and the OOH Index with mortality for those with the same RGP in the group of chronic conditions. All analyses were conducted using Stata (version 16.1).

## Results

**Figure 1.**
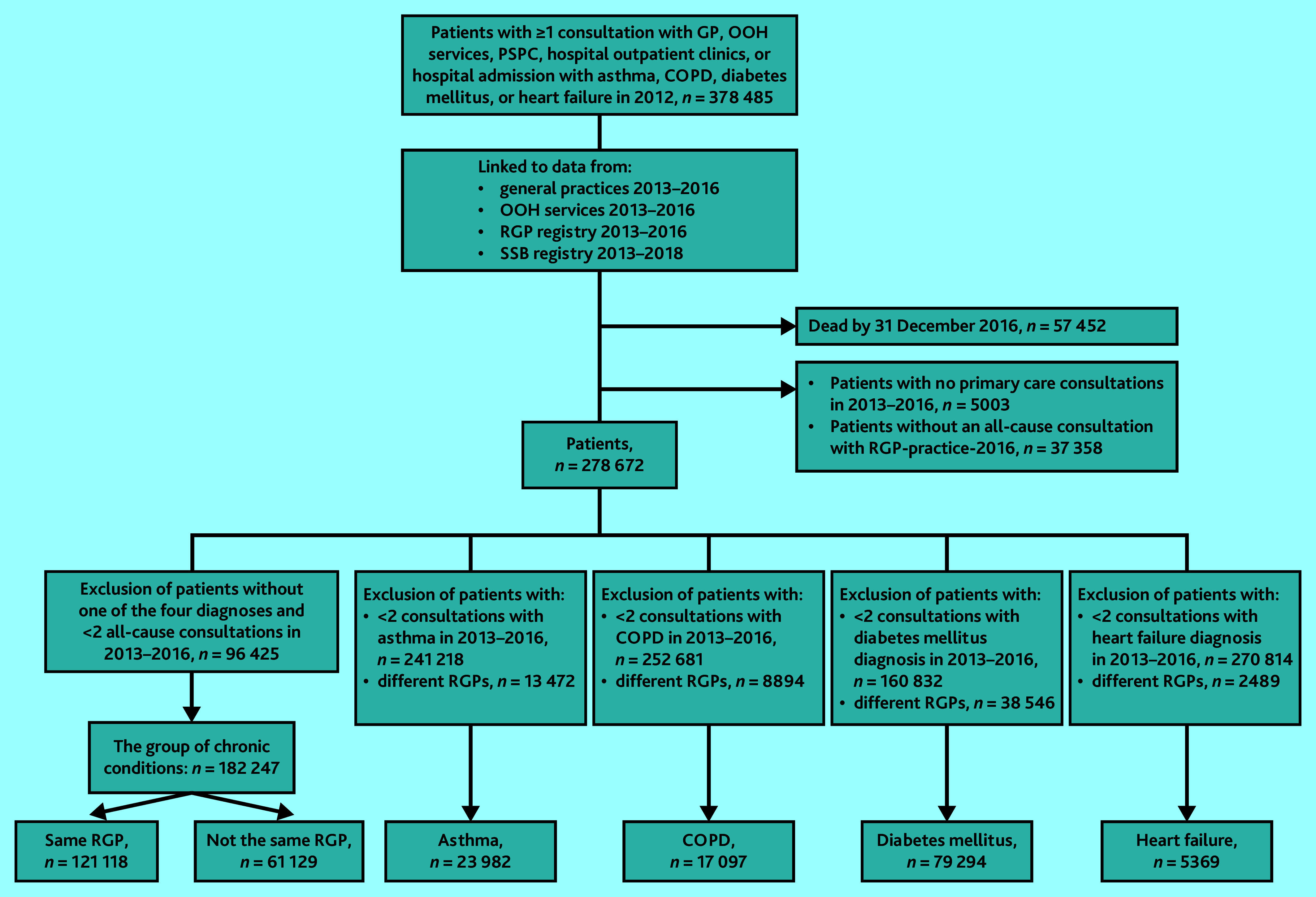
Flow chart showing the inclusion and exclusion process for the five study populations: asthma, chronic obstructive pulmonary disease, diabetes mellitus, heart failure, and the group of chronic conditions. Patients were identified in 2012 and have ≥2 disease-related consultations in the four diagnosis populations and ≥2 all-cause consultations in the group of chronic conditions. COPD = chronic obstructive pulmonary disease. OOH = out of hours. PSPC = private specialist with public contracts. RGP = regular GP. SSB = Statistics Norway.

Application of inclusion and exclusion criteria led to the five study populations shown in Figure 1. Patients’ characteristics of these populations are shown in Table 1. Regarding CoC, both UPC^disease^ and UPC^all^ were generally high (mean ≥0.70) for all four diagnoses populations (Supplementary Table S1).

**Table 1.** Patient characteristics in the study populations with asthma, COPD, diabetes mellitus, heart failure, and the group of chronic conditions which combines these diagnoses, 2013–2016^a^

**Characteristic**	**Asthma**		**COPD**		**Diabetes mellitus**		**Heart failure**		**Group of chronic conditions**			

									**Same RGP**		**Changed RGP**	

	***n***	**%**	***n***	**%**	***n***	**%**	***n***	**%**	***n***	**%**	***n***	**%**
**Total**	23 982	100	17 097	100	79 294	100	5369	100	121 118	100	61 129	100

**Sex**												
Female	14 045	58.6	8540	50.0	34 827	43.9	2340	43.6	57 500	47.5	30 377	49.7
Male	9937	41.4	8557	50.0	44 467	56.1	3029	56.4	63 618	52.5	30 752	50.3

**Age group, years**												
Mean age (SD)	48 (25)		73 (10)		68 (14)		80 (12)		65 (19)		63 (20)	
<65	16 460	68.6	2994	17.5	28 549	36.0	567	10.6	47 554	39.3	26 674	43.6
≥65	7522	31.4	14 103	82.5	50 745	64.0	4802	89.4	73 564	60.7	34 455	56.4

**Centrality Index**												
1 (most urban)	4731	19.7	2587	15.1	14 403	18.2	907	16.9	21 816	18.0	9278	15.2
2	6158	25.7	4409	25.8	19 049	24.0	1319	24.6	29 789	24.6	13 361	21.9
3	6590	27.5	4763	27.9	21 717	27.4	1552	28.9	33 333	27.5	14 837	24.3
4	4469	18.6	3418	20.0	15 136	19.1	955	17.8	23 076	19.1	12 665	20.7
5	1587	6.6	1415	8.3	6810	8.6	475	8.8	9928	8.2	7235	11.8
6 (most rural)	447	1.9	504	2.9	2179	2.7	161	3.0	3175	2.6	3636	5.9
Missing	0	0.0	1	0.01	0	0.0	0	0.0	1	0.001	117	0.2

**Educational level**												
Low	6383	26.6	7423	43.4	26 673	33.6	2096	39.0	40 545	33.5	21 532	35.2
Medium	8226	34.3	7990	46.7	37 502	47.3	2437	45.4	54 104	44.7	25 590	41.9
High	4739	19.8	1552	9.1	14 079	17.8	788	14.7	20 670	17.1	10 494	17.2
Missing	4634	19.3	132	0.8	1040	1.3	48	0.9	5799	4.8	3513	5.7

**Comorbidities^b^**												
0	17 506	73.0	249	1.5	523	0.7	175	3.3	18 413	15.2	10 256	16.8
1	4844	20.2	9917	58.0	57 764	72.8	2500	46.6	73 726	60.9	35 128	57.5
2	1329	5.5	5084	29.7	16 758	21.1	1910	35.6	22 943	18.9	11 986	19.6
≥3	303	1.3	1847	10.8	4249	5.4	784	14.6	6036	5.0	3759	6.1

**Deaths during 2017–2018**	493	2.1	2613	15.3	4611	5.8	1443	26.9	8401	6.9	4309	7.0

a *Patients in the four diagnosis groups had the same RGP during the observation period 2013–2016.*

b *Number of comorbidity groups based on ICPC Morbidity Index.^28^ Data are* n *(%) apart from for the Mean age row. COPD = chronic obstructive pulmonary disease. RGP = regular GP. SD = standard deviation.*

In the group of chronic conditions, as might be expected the mean UPC^all^ was lower (0.65) for those who had changed personal RGPs than for those with the same personal RGP (0.78) in the study period (Supplementary Table S2).

### CoC with personal RGP and mortality

**Figure 2.**
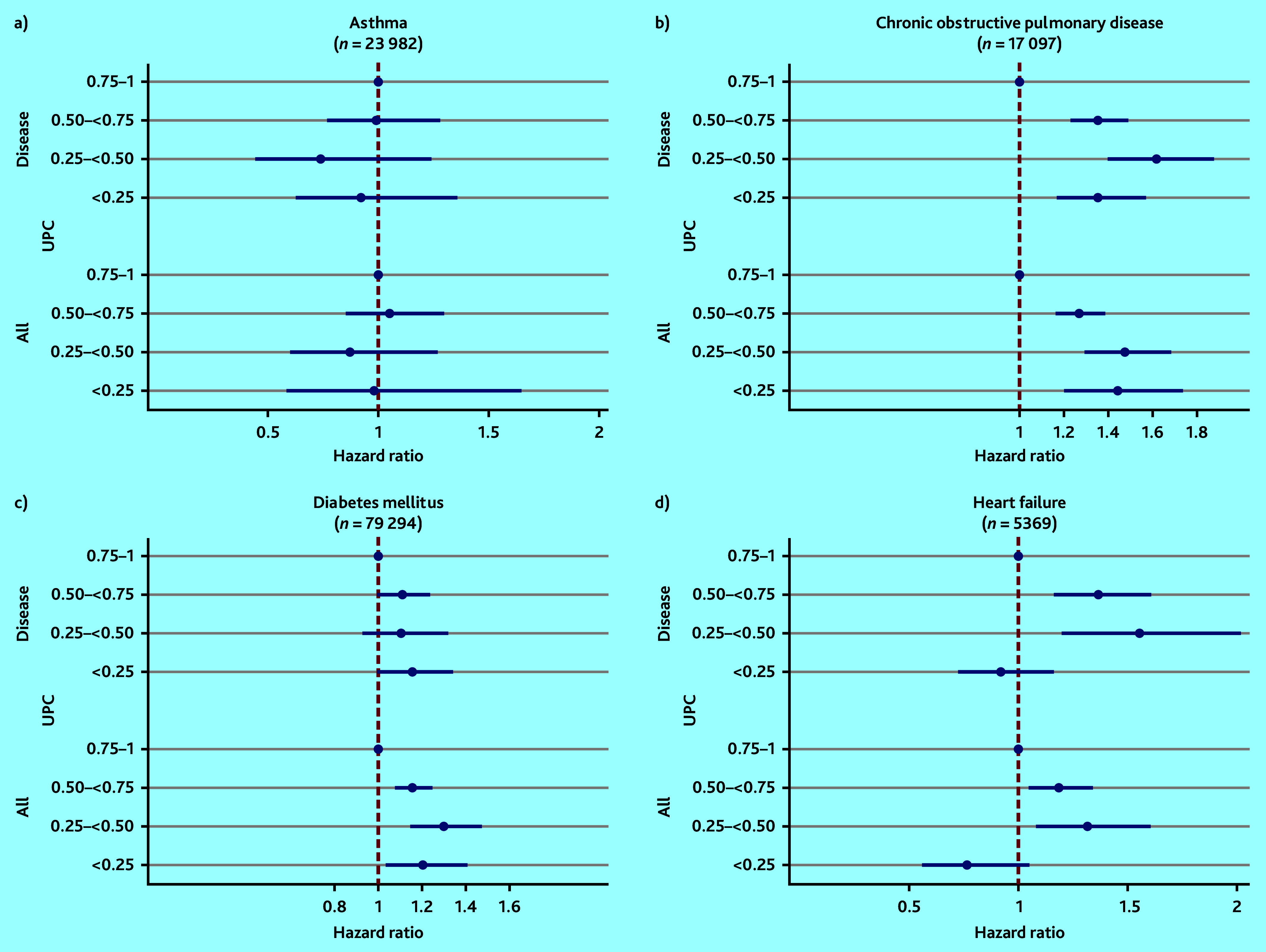
Mortality associated with overall UPC (UPC^all^) and disease-related UPC (UPC^disease^) for patients a) with asthma; b) chronic obstructive pulmonary disease; c) diabetes mellitus; and d) heart failure, adjusted for age (continuous), sex, Centrality Index, and comorbidity groups^a^ (based on ICPC Morbidity Index^b^). The horizontal lines on x-axis present the 95% confidence interval and the hazard ratio. Reference category is the group with patients with the highest UPC (0.75–1.00). ^a^The analyses for UPC^all^ are not adjusted for comorbidity. ^b^ Number of comorbidity groups based on ICPC Morbidity Index.^28^ ICPC = International Classification of Primary Care. UPC = Usual Provider of Care Index (where 1 = all consultations by the RGP).

In the COPD and heart failure populations, using the highest UPC (0.75–1) as reference, the risk of death was significantly higher in all the groups with lower UPC^all^ and UPC^disease^, apart from the groups with the lowest UPCs in the heart failure population (Figure 2b and 2d). For instance, patients with COPD with UPC^disease^ <0.25 had 47% increased risk of death within 2 years (hazard ratio [HR] 1.47, 95% confidence interval = 1.22 to 1.64) compared with those with UPC^disease^ ≥0.75.

For patients with diabetes, all the groups with lower UPC^all^ were associated with increased mortality in the adjusted analyses compared with the highest UPC^all^, although there was no significant association between mortality and UPC^disease^ (Figure 2c and Supplementary Tables S3 and S4).

In patients with asthma, no statistically significant association was found between lower groups of UPC and mortality in adjusted analyses (Figure 2a, Supplementary Tables S3 and S4).

**Figure 3.**
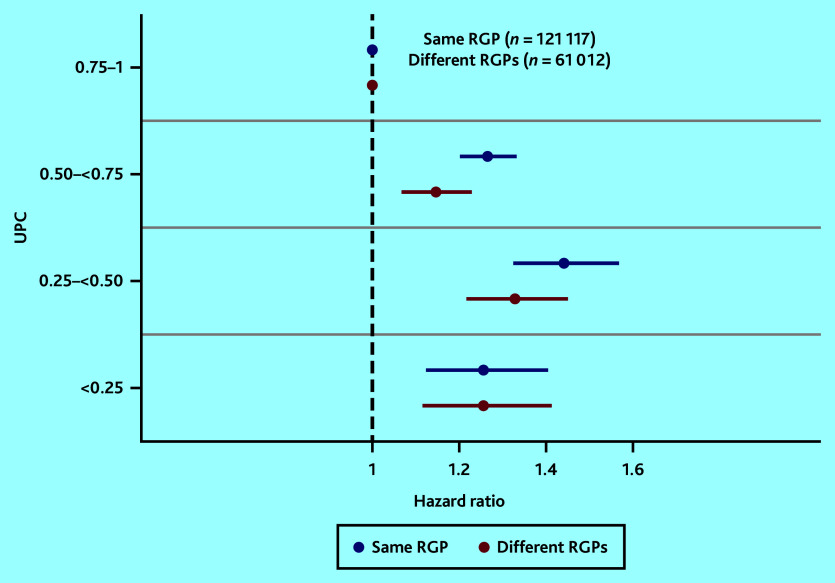
Mortality associated with UPC^all^ for patients in the group of chronic conditions, both with the same and different RGPs, adjusted for age (continuous), sex, and Centrality Index. The reference category is the group with patients with the highest UPC (0.75–1). RGP = regular GP. UPC = Usual Provider of Care Index (where 1 = all consultations by the RGP).

Figure 3 shows adjusted analyses of the associations between UPC^all^ and mortality for the group of chronic conditions, stratified by patients with the same RGP and those who had changed RGP. An increasing risk of death with decreasing UPC^all^ was found in both strata, barring the group with the lowest UPC (0–0.25) (Supplementary Table S5).

By decreasing RGP continuity, utilisation of other GPs increased substantially, whereas the use of OOH services did not change much (Supplementary Table S5). The group with lowest UPC stands out compared with other levels of UPC by having few consultations with the named RGP (on average 3 of 23 consultations).

In the combined group of all four chronic conditions, analysis in patients with the same RGP, the HR for death were higher for all groups of the Other GPs Index compared with the reference group (Other GPs Index <0.1), apart from the second highest category with an Other GPs Index = 0.3–0.4 (adjusted analyses), with no dose–response trend. Regarding the OOH Index, the HR for death increased gradually from 1.23 (95% CI = 1.16 to 1.30) to 2.16 (95% CI = 2.00 to 2.32) compared with the OOH Index reference group (<0.05) (Table 2).

**Table 2.** The association between the proportion of consultations with other GPs and OOH services during 2013–2016 and mortality for patients in the group of chronic conditions with the same RGP during the observation period

**Index**	**Patients**			**Mortality**			

	**Total**		**Died in 2017–2018**	**Crude**		**Adjusted^a^**	

	***n***	**%**	**%**	**HR**	**95% CI**	**HR**	**95% CI**
**Other GPs Index^b^**	121 118	100	6.9				
<0.1	64 167	53.0	6.8	Reference		Reference	
0.1 to <0.2	22 976	19.0	7.7	**1.13**	**(1.07 to 1.20)**	**1.12**	**(1.06 to 1.18)**
0.2 to <0.3	12 929	10.7	6.8	0.99	(0.92 to 1.07)	**1.10**	**(1.02 to 1.18)**
0.3 to <0.4	7350	6.1	6.5	0.94	(0.86 to 1.04)	1.07	(0.97 to 1.18)
≥0.4	13 696	11.3	6.5	0.95	(0.88 to 1.02)	**1.12**	**(1.04 to 1.21)**

**OOH Index^b^**	121 118	100	6.9				
<0.05	73 437	60.6	5.7	Reference		Reference	
0.05 to <0.1	21 918	18.1	8.0	**1.41**	**(1.34 to 1.49)**	**1.23**	**(1.16 to 1.30)**
0.1 to <0.15	11 529	9.5	8.5	**1.50**	**(1.40 to 1.61)**	**1.39**	**(1.30 to 1.49)**
0.15 to <0.2	5313	4.4	10.2	**1.80**	**(1.65 to 1.97)**	**1.71**	**(1.56 to 1.87)**
≥0.2	8921	7.4	10.2	**1.81**	**(1.68 to 1.94)**	**2.16**	**(2.00 to 2.32)**

a *Adjusted for age (continuous), sex, Centrality Index, and comorbidity groups based on ICPC Morbidity Index^28^ in a Cox regression model. Results in bold are significant.*

b *Both the Other GPs Index and OOH Index have a value 0 to 1 and are categorised in 5 groups: Other GP Index (<0.1, 0.1–<0.2, 0.2–<0.3, 0.3–<0.4, and ≥0.4) and OOH Index (<0.05, 0.05–<0.1, 0.1–<0.15, 0.15–<0.2, and ≥0.2). HR = hazard ratio. ICPC = International Classification of Primary Care. OOH = out of hours. RGP = regular GP.*

## Discussion

### Summary

This study investigated the association between RGP continuity and mortality for patients with four selected chronic conditions. Both lower disease-related and overall RGP continuity were associated with higher mortality, although not always with a clear trend, for patients with diabetes, COPD, and heart failure. No association was found for patients with asthma. There were no statistically significant differences in the associations comparing patients with the same RGP with those who had changed their named RGPs, defined as the RGP at the time of consultation as the usual provider. Breaches in continuity by utilisation of OOH services increased the risk of death more than use of other GPs.

### Strengths and limitations

Obtaining data from linking several national registries including the whole population limits the selection bias, and use of all consultations in a rather long observation period of 4 years provides a good foundation for estimating CoC. It may be argued that two consultations in 4 years is low for measuring RGP continuity, but choosing a higher number of consultations for inclusion could lead to small strata in the disease-specific populations and could introduce bias by omitting patients with little use of care and less serious disease. However, as >75% of patients had ≥4 consultations during the observation period, the authors believe this study provides adequate continuity measurements.

Annual updates from the RGP registry may introduce some inaccuracies about whether consultations are with the actual RGP at a specific date. The data dates from 2012 to 2018, but the authors strongly believe that the current findings are valid as there has been no organisational changes in the RGP list system. Since the COVID-19 pandemic the consultation rate, including the use of electronic consultations, has somewhat increased, but most contacts are still in-person consultations^29^ and the authors believe that the changes during the pandemic do not significantly have an impact on CoC and its consequences. The current dataset lacks information on risk factors such as smoking that could be a co-predictor for mortality as well as sociodemographic factors such as ethnicity that could be relevant covariates regarding care-seeking behaviour.

To the best of the authors’ knowledge, there are no studies on mortality and RGP continuity for both chronic and overall care, including data on breaches of CoC, for example, same or changed RGP. The current study adds important insights to the CoC literature.

### Comparison with existing literature

For CoC and mortality, the current findings show high disease-related and overall continuity for patients with chronic diseases in Norway, supporting the current authors’ earlier studies.^23^^,^^30^ Both UPC^disease^ and UPC^all^ are very high in the current study compared with other countries.^12^ The majority of the patients with COPD, diabetes, and heart failure who had high RGP continuity also had lower mortality, indicating that these patients benefit from high RGP continuity both for their chronic conditions and their overall healthcare needs. This suggests that care should be designed to ensure high overall continuity and not be planned solely for chronic disease management.

The association between continuity and mortality was not significant in adjusted analyses for patients with asthma. This can be explained by a younger population (mean age 48 years) with low mortality (2.1%, *n* = 493/23 982), in line with another study.^23^ Unexpectedly, the highest mortality was not in the groups with the lowest CoC. One explanation might be that patients with a UPC <0.25 mostly use other GPs or long-term locum doctors who work on their RGP’s lists, and thus *de facto* have a continuous usual provider and therefore achieve the benefits of a high CoC despite a low UPC in this study. This assumption is supported by a recent report regarding patient lists with no RGP responsible, showing a UPC around 0.60 when defining the most seen GP as the usual provider.^31^

For discontinuity of care and mortality, the current study found no significant differences for the association between CoC and mortality comparing those with the same RGP and those who had changed RGP during these 4 years, despite such a breach in personal continuity. This was unexpected and in contrast to the study by Sandvik *et al* that showed a strong association between having the same RGP and reduced mortality and acute hospital admissions,^6^ this was also supported by another Norwegian study, showing increased acute hospital admissions in older age groups associated with RGP discontinuity.^32^ The authors of the current study believe that patients who change their RGP are a heterogeneous group and probably many of them become listed with a new RGP in the same practice. Besides, the mean UPC was rather high in both groups, indicating a loyalty to the RGP responsible. The current findings thus support that a system with RGPs with personal lists provides high informational and management continuity, which subsequently reduces the negative effects on patient outcomes from breach in personal continuity.

A high OOH Index has a much stronger association with increased mortality compared with the Other GPs Index. This may reflect more serious disease, but most contacts with OOH services are not emergency related,^33^ and this finding supports the benefit of informational and management CoC as discussed above.

### Implications for research and practice

The prevalence of chronic diseases is rising^34^ and the current findings have important policy implications regarding future planning of general practice, emphasising CoC as a quality indicator. However, CoC does not evolve only over certain disease-related consultations and it takes time for doctors to build a relationship with ‘accumulated knowledge’ about their patients.^35^ The results from the current study suggest that RGPs should organise their practices in future to achieve high CoC for their patients with chronic disease by including all-cause consultations, rather than solely focusing on chronic disease-related consultations. Another recommendation is that policymakers and healthcare planners implement measures such as RGP personal list systems to provide and maintain CoC concerning all consultations in primary care. The current study also revealed that different types of breaches in CoC have varying effect on mortality. Further research on the impact of having a named RGP, even if this provider may change occasionally, is required. Also, the magnitude of effects from informational and management continuity compared with personal CoC needs further investigation.

## Data

Data were provided by the Norwegian Health Economics Administration (HELFO), Norwegian Patient Registry, and Statistics Norway. Due to restrictions by the Norwegian Data Protection Authority, the data cannot be shared publicly.
